# Psychophysiological Stress Response in an Underwater Evacuation Training

**DOI:** 10.3390/ijerph17072307

**Published:** 2020-03-30

**Authors:** Marta Vicente-Rodríguez, Juan Pedro Fuentes-Garcia, Vicente Javier Clemente-Suárez

**Affiliations:** 1 Psychophysiological Research Group, European University of Madrid, Tajo Street, s/n, 28670 Madrid, Spain; marta_v_fuenla@hotmail.com; 2 Faculty of Sports Sciences, University of Extremadura, Av. de la Universidad, S/N, 10003 Cáceres, Spain; jpfuent@unex.es; 3 Faculty of Sports Sciences, Universidad Europea de Madrid, Tajo Street, s/n, 28670 Madrid, Spain; 4 Grupo de Investigación en Cultura, Educación y Sociedad, Universidad de la Costa, 080002 Barranquilla, Colombia

**Keywords:** stress, military, aircrew, accident, cortical arousal, heart rate variability

## Abstract

Background: This research aimed to analyze the psychophysiological stress response of air crews in an underwater evacuation training. Materials and Methods: We analyzed in 36 participants (39.06 ± 9.01 years) modifications in the rating of perceived exertion (RPE), subjective stress perception (SSP), heart rate (HR), blood oxygen saturation (BOS), cortical arousal (critical flicker fusion threshold, CFFT), heart rate variability (HRV), spirometry, isometric hand strength (IHS), and short-term memory (ST-M) before and after an underwater evacuation training. Results: The maneuver produced a significant (*p* ≤ 0.05) increase in the SSP, RPE, Mean HR and maximum HR (Max HR), and a decrease in minimum HR (Min HR) and HRV. Conclusion: An underwater evacuation training produced an increase in the sympathetic nervous system modulation, elevating the psychophysiological stress response of the air crews, not negatively affecting their cortical arousal.

## 1. Introduction

Military deployments in current conflicts zones are one of the most stressful and demanding situations or work experiences than a person can face. The dangerous, unpredictable, and unknown nature of military operations makes especially important cognitive processes such as perception, decision-making, processing information, attention, memory, as well as physical skills such as strength, flexibility, and endurance [1,2,3]. The psychophysiological modification observed in different military actions by previous research highlighted the important need to show a deep analysis of psychophysiological parameters involved in stress situations and for the urgent creation of personalized training, that is more realistic and adapted to their requirements [4,5,6,7]. In the context of actual military missions, adding to the chronic exposure to this stressful environment increases the probability of developing mental pathologies such as post-traumatic stress disorder (PTSD), a syndrome characterized by the development of specific symptoms after direct or indirect exposure to traumatic events [8,9].

Specifically in air force crews, previous research found how exposure to normobaric hypoxia training produced psychophysiological changes in sympathetic nervous system modulation, cortical arousal levels, and increments in stress hormones such as cortisol and testosterone levels among others [3,10,11,12,13,14,15,16,17]. In this line, research on paratrooper also showed how high-altitude jumps (High Altitude Low Opening and High Altitude High Opening) produced an increase in the physiological response and sympathetic modulation in both jumps, according to the stress responses theory exposed by Selye [18], and a decrease in cortical arousal, which is a symptom of central nervous system (CNS) fatigue only in the HAHO jump, showing that both types of jumps are stressful stimuli for the paratroopers; besides, tactical automatic parachute jumps produced an increase in cortisol hormone production that is related with the activation of the fight–flight response that increases the sympathetic autonomic modulation [14,17,18,19].

All the crews and personal who fly in aircrafts are exposed to having an air accident and this event could happen over water. The survival of the personnel in this extreme situation depends to a great extent on the previous underwater evacuation training conducted. However, very little is known about the physical and mental requirements of this specific and demanding training to improve it, and given its importance, it is very relevant to make this training more operational and specific to allow not only a better survival in air accidents in water areas but a better adaptation and recovery in this extreme situation. For this reason, we conducted the present research with the aim of analyzing the psychophysiological response of air crews in an underwater evacuation training. We hypothesized that underwater evacuation training would decrease cortical arousal of participants due to the increase in the sympathetic autonomic modulation, a fact that would negatively affect cortical functions such as memory.

## 2. Methods

### 2.1. Participants

In the present work we analyzed 36 participants, 26 men and 10 women, (39.06 ± 9.01 years; 174.2 ± 7.35 cm; 76.4 ± 11.98 kg; 16.72 ± 9.34 years of service; 13.15 ± 14.32 months in mission; 182.28 ± 30.50 flying hours). Before participation, experimental procedures were explained to all the participants, who gave their voluntary written informed consent in accordance with the Declaration of Helsinki. All the procedures were approved by the University Ethics Committee. The entire research procedure was conducted by soldiers with the standard flying suit and boots, portable oxygen cylinder, life vest, helmets, and earphones to simulate the equivalent operating equipment for this type of action. None of the participants had previous or recent experience in these underwater simulations.

### 2.2. Research Design, Instrumentation, and Study Variables

A pre–post intervention was conducted analyzing the following psychological and physiological variables previous to and immediately after an underwater evacuation training.

#### 2.2.1. Psychological Variables

1. Rating of perceived exertion (RPE), Borg 6–20 scale [20].
2. Stress subjective perception (SSP) on a 1–100 scale [11].
3. Short-term memory (ST-M). A three digit number was shown to the participants for 1 s. After 5 s, they were asked to remember the number and to say it backward [11].

#### 2.2.2. Biosensor Equipment

1. Blood oxygen saturation (BOS) and heart rate (HR) were measured by a finger pulse-oximeter system (PO 30 Beurer Medical) [14].
2. Cortical arousal and fatigue of the CNS was measured by the Lafayette Instrument critical flicker fusion threshold (CFFT) (Model 12,021) by the average of five incremental tests (20–100 Hz) according to previous authors [5,14,21].
3. Isometric hand strength (IHS) was measured by a grip dynamometer (Takei Kiki Koyo, Japan) [14].
4. Spirometry: Forced vital capacity (FVC), volume exhale at the end of the first second of forced expiration (FEV1), and the peak expiratory flow (PEF) were measured using a QM-SP100 (Quirumed, Valencia Spain) spirometer in a maximum inhale–exhale cycle.

Finally, the heart rate (HR) and heart rate variability (HRV), which refers to the autonomic modulation, were recorded with a Polar V800 heart rate monitor with RR measurement function (POLAR, Finland) and posteriorly analyzed by the Kubios HRV standard 3.2.0 software (University of Kuopio, Kuopio, Finland) [14]. The parameters analyzed were as follows:

- Temporal domain: mean HR; minimum HR (Min HR); maximum HR (Max HR); square root of the mean value of all sums of square differences of all R–Rs following intervals (RMSSD); the percentage of differences between normal adjacent R–R intervals greater than 50 ms (pNN50);
- Frequency domain: low-frequency band (LF); high-frequency band (HF); a ratio of low frequency to high frequency (LF/HF ratio);
- Nonlinear domain: sensitivity of the short-term variability (SD1); sensitivity of the long-term variability (SD2).

### 2.3. Statistical Analysis

Statistical analysis was performed with the SPSS 21.0 statistical program. The descriptive statistics used to report the results were the mean ± standard deviation (SD). Normality of the sample was determined with the Kolmogorov–Smirnov test as all the variables presented a parametric distribution. A repeated-measures ANOVA was conducted to test differences in the evaluation moment analyzed. The effect size was calculated by Cohen’s D. The significance level was *p* ≤ 0.05.

### 2.4. Description of the Underwater Evacuation Training

**Figure 1:**
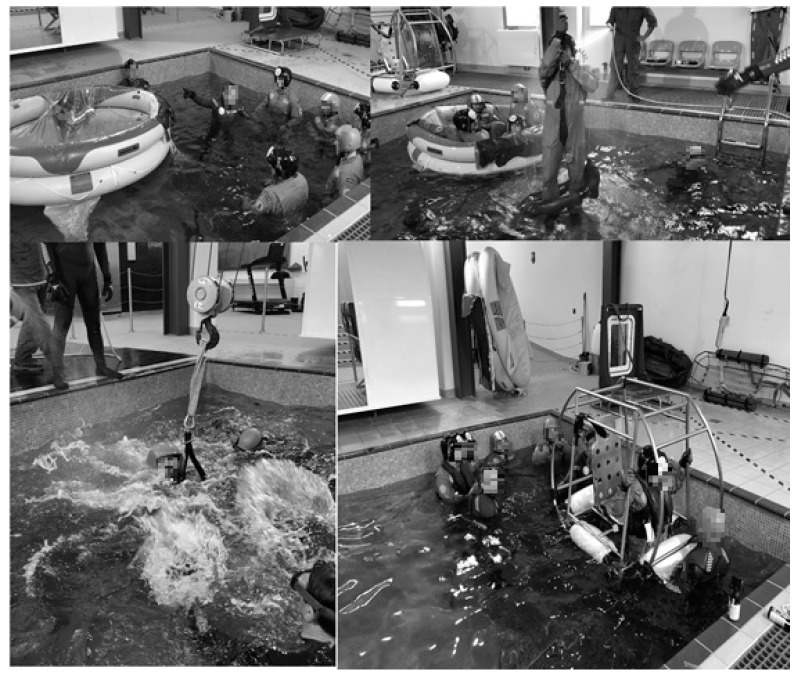
Four moments of the underwater evacuation training.

The underwater evacuation training simulated the demands of an aircraft accident, being conducted for two hours in a swimming pool in the presence of two instructors and two water assistants (Figure 1). The characteristics of the swimming pool were: 4 × 4 × 3 m; water characteristic: chlorine 2; pH 7.5; turbidity values 0.13; temperature: 26 °C. Participants conducted the following exercises:

1. Apnea training using the portable oxygen cylinder
  Duration: 30 min.

With the aim of exemplify the difficulty to get and control the limited oxygen in an extreme situation, the participants rehearsed apnea training under water using a portable oxygen cylinder and staying submerged for a few minutes while doing different positions such as getting vertically submerged using the pool to keep that position.

2. Evacuation of the submerged aircraft through a window
  Duration: 30 min.

Using a metallic platform underwater to keep participants standing during the explanation and using a window to simulate an aircraft submerging, the participants had to unlock and open the window and pass through it one by one while the instructors moved the instrument to simulate aircraft movement under water.

3. Rescue training of a raft and rescue in water from a helicopter
  Duration: 30 min.

The participants had to turn over the raft looking for and using the oxygen tank in one of the sides of the raft, while the instructors simulated sea conditions, and try to jump inside it one by one. After that, they had to jump off the boat and simulate a rescue in the water from a helicopter using a harness while the instructors kept simulating the sea conditions.

4. Underwater evacuation during the turn of the ship using the portable oxygen cylinder
  Duration: 30 min.

The participants had to sit in the instrument and fasten the seat belt. They remained in this position, crossing their arms in a safety movement, while the instructors turned the ship, and while controlling the air and using the portable oxygen device as in the first exercise, they had to get out of the submerged ship by unlocking the window.

## 3. Results

The results are reported as mean ± SD. The pre–post results of the psychophysiological variables studied are shown in Table 1. SSP and RPE presented a significant increase and no significant differences were observed in the other pre–post variables analyzed.

The autonomic response explained by the HRV measured before, during, and after the maneuver is shown in Table 2. Mean HR presented a significant increase during the maneuver and in the post moment in comparison with the pre moment. Min HR and LF/HF Ratio presented a significant decrease during the maneuver in comparison with pre values and a significant increase in post values. Max HR, RMSSD, pNN50, HF, SD1, and SD2 variables showed a significant increase during the maneuver and a significant decrease in post values. LF presented a significant decrease during the maneuver in comparison with pre values and a significant increase in post values.

## 4. Discussion

This study aimed to analyze the psychophysiological modifications of air crews in an underwater evacuation training. The initial hypothesis was partially fulfilled since an increase in the sympathetic autonomic modulation of the air crews was monitored, but there was no significant decrease in the participants’ cortical arousal. 

We found a significant increase in SSP and RPE values between pre–post intervention that did not negatively affect physiological parameters such as FVC, FEV1, PEF, IHS, BOS, and HR, or psychological parameters such as CFFT or ST-M. The low activation of the psychophysiological response of this maneuver was more contrary than other studies in the military population where there was no rest between the different situations that compose the maneuver [5,22,23]. The results obtained evidenced the soldiers’ perception of the maneuver as an elicitant and aversive stimuli, which would affect cognitive processes, but the breaks between underwater exercises could lead participants to a physical recuperation between them. In other research conducted with military populations, similar to the present study, authors found a decreased tendency in cortical arousal and a rise in perceived effort and stress, showing that the specific stress response depends on the intrinsic characteristic of the maneuver [17,24].

We measured an anticipatory anxiety response reflected by the low values of RMSSD, pNN50, SD1, and SD2, and the high values of LF variables before the maneuver. This initial stress response provoked by the activation of the fight–flight system prepares the organism for an eliciting activity being mediated by the level of uncertainty and the physical and mental requirements of the task [2,3,25]. Previous studies also found that personal experience modulates the stress perception, reducing the negative impact of large sympathetic modulation evaluated on ST-M, CFFT, BOS, and HR [1,7,15,17].

We also measured a significant increase in HR values (Mean HR, Min HR, and Max HR) after the maneuver. According to previous studies, this physiological activation is related with the stress produced during the intervention, a result in line with the significant increase in SSP and RPE and the aforementioned increase in the sympathetic nervous system modulation [2,3,12,16,25]. However, despite the significant increase in HR, the values reached were lower than those in other military maneuvers such as asymmetrical, symmetrical, melee, urban, and underground combat, NBQ maneuvers, tactical, HALO and HAHO parachute jumps, air mobile protection team actions, or checkpoint actions [4,5,12,15,16,21,22,23,26,27,28,29]. This result highlighted the lower cardiovascular requirements of underwater evacuation training, but we have to take in account the bradycardia effect of cold-water exposure, a fact that could interfere in the cardiovascular response, causing it to be lower than that in other military maneuvers [30]. The cardiovascular response was more related with that obtained in normobaric hypoxia training in military aircrews, helicopter aircrew in night and instrument flights, or fighter reactor pilots in combat flying maneuvers, probably due to the lower muscular requirements of the first one and the influence of G forces in the second one, factors that made possible a cardiovascular response similar to the one evaluated in the present research [11,13,31]. The average HR evaluated was consistent with an aerobic physical activity workload, presenting some demanding moments (as we show in maximal HR) where anaerobic demands increase due to the high and intense demands of the different activities carried out in the underwater training [32,33,34]. Regarding the autonomic modulation domain analysis, we found that the variables of the HRV temporal domain (HR, Min HR, Max HR, RMSSD, and pNN50) and the nonlinear domain (SD1 and SD2) analyzed in the present research were sensitive for discriminating the autonomic response, while in other studies not all the variables of the frequency domain such as HF have shown such sensibility [35]. It seems that depending on the participants and evaluation context, different HRV parameters showed different sensitivity, more research in this line is needed to determine the most sensitive HRV analysis domain. 

### 4.1. Limitations of the Study and Future Research Lines

The principal limitations of the research were the absence of cortisol levels measurements, as well as, the evaluation of blood lactate concentrations as measurements to obtain direct stress and physical exertion information in the simulation. Furthermore, the lack of an electroencephalogram as a direct measurement of cortical arousal systems is considered a limitation. For future research, we propose the analysis of the psychophysiological stress response in different aircrew, as well as, analysis of the effect of experience in the psychophysiological stress response and operability of the aircrew.

### 4.2. Practical Applications

This study improves the knowledge of psychophysiology in extreme environments such as aviation accidents and underwater rescue, providing a basis to improve safety and specific preparation for air crews in emergencies, as well as, to reduce the risk of physical injuries or mental pathologies, which can negatively affect not only the success of the operation but also their long-term quality of life. The information obtained in the present research has a direct application in combination with previous research for designing specific training for helicopter and aircraft crew preparation [31,36,37].

## 5. Conclusions

An underwater evacuation training produced an increase in the sympathetic nervous system modulation and did not negatively affect cortical arousal and strength manifestations.

## Figures and Tables

**Table 1:** Changes mean and standard deviation (M ± SD) in psychophysiological variables pre- and post-maneuver.

Variables	Pre-Maneuver	Post-Mmaneuver	F	*p*	Size Effect
SSP	22.17 ± 23.04	56.25 ± 26.36	59.668	0.000	1.48
RPE	6.36 ± 1.10	12.47 ± 2.99	179.907	0.000	5.55
IHS (N)	46.08 ± 12.60	46.58 ± 12.56	0.570	0.455	0.04
FVC (mL)	4.25 ± 1.18	4.61 ± 0.96	3.482	0.070	0.31
FEV1 (mL)	3.54 ± 1.07	3.78 ± 0.77	2.417	0.129	0.22
PEF (mL)	9.54 ± 2.72	9.68 ± 2.28	0.163	0.689	0.05
BOS (%)	96.31 ± 1.80	96.17 ± 2.49	0.064	0.801	−0.08
HR (bpm)	80.50 ± 14.88	78.69 ± 18.05	0.456	0.504	−0.12
S-TM	1.00 ± 0.00	1.00 ± 0.00	—	—	—
CFFT (Hz)	39.35 ± 3.59	37.94 ± 3.70	2.847	0.100	−0.39

SSP—stress subjective perception; RPE—rating of perceived exertion; IHS—isometric hand strength; FVC (forced vital capacity), FEV1 (volume exhale at the end of the first second of forced expiration), PEF (peak expiratory flow)—spirometry; BOS—blood oxygen saturation; HR—heart rate; ST-M—short-term memory; CFFT—critical flicker fusion threshold.

**Table 2:** Changes (M ± SD) in the autonomic response (HRV) pre-, during, and post-maneuver.

Variables	Pre-Maneuver (1)	During Maneuver (2)	Post-Maneuver (3)	F	*p*	Moments Comparison
Mean HR (bpm)	88.45 ± 14.69	91.14 ± 10.67	97.68 ± 13.36	12.608	0.000	1 < 3; 2 < 3; 3 > 2 > 1
Min HR (bpm)	67.15 ± 12.46	54.56 ± 16.03	69.13 ± 11.55	0.495	0.000	1 > 2; 2 < 1< 3; 3 > 2
Max HR (bpm)	124.68 ± 30.65	174.16 ± 53.79	140.93 ± 37.31	15.008	0.000	1 < 2; 2 > 3 > 1; 3 < 2
RMSSD (ms)	33.11 ± 18.73	43.96 ± 17.02	35.71 ± 18.93	12.197	0.000	1 < 2; 2 > 3 > 1; 3 < 2
pNN50 (%)	7.42 ± 7.28	10.02 ± 5.62	6.75 ± 5.23	9.516	0.001	2 > 3; 3 > 2
LF (n.u.)	79.08 ± 11.63	73.06 ± 9.11	76.15 ± 10.09	4.208	0.023	1 > 2; 2 < 1
HF (n.u.)	20.85 ± 11.58	26.83 ± 9.04	23.75 ± 10.02	4.189	0.024	1 < 2; 2 > 1;
Ratio LF/HF	5.27 ± 3.25	3.50 ± 3.00	4.40 ± 3.45	3.422	0.044	1 > 2; 2 < 1;
SD1 (ms)	23.50 ± 13.46	30.94 ± 12.11	25.31 ± 13.49	12.047	0.000	1 < 2; 2 > 3 > 1; 3 < 2
SD2 (ms)	57.44 ± 22.24	62.89 ± 16.80	55.25 ± 18.72	6.439	0.004	2 > 3; 3 < 2;

Mean HR—mean heart rate; Min HR—minimum heart rate; Max HR—maximum heart rate; RMSSD—square root of the mean of the sum of the squared differences between adjacent normal R–R intervals; pNN50—percentage of differences between normal adjacent R–R intervals greater than 50 ms; LF—low frequency; HF—high frequency; Ratio LF/HF—ratio of low frequency to high frequency; SD1—standard deviation of the scattergram 1; SD2—standard deviation of the scattergram 2; n.u.—normalized unit.

## References

[1] Delgado-Moreno R., Robles-Pérez J.J., Aznar-Laín S., Clemente-Suárez V.J. (2019). Effect of Experience and Psychophysiological Modification by Combat Stress in Soldier’s Memory. J. Med. Syst..

[2] Curiel-Regueros A., Fernández-Lucas J., Clemente-Suárez V.J. (2019). Effectiveness of an applied high intensity interval training as a specific operative training. Physiol. Behav..

[3] Sánchez-Molina J., Robles-Pérez J.J., Clemente-Suárez V.J. (2017). Effect of parachute jump in the psychophysiological response of soldiers in urban combat. J. Med. Syst..

[4] Clemente-Suarez V.J., Robles-Perez J.J. (2013). Mechanical, physical, and physiological analysis of symmetrical and asymmetrical combat. J. Strength Cond. Res..

[5] Clemente-Suarez V.J., Palomera P.R., Robles-Pérez J.J. (2018). Psychophysiological response to acute-high-stress combat situations in professional soldiers. Stress Health.

[6] Delgado-Moreno R., Robles-Pérez J.J., Clemente-Suárez V.J. (2017). Combat stress decreases memory of warfighters in action. J. Med. Syst..

[7] Tornero-Aguilera J.F., Robles-Pérez J.J., Clemente-Suárez V.J. (2017). Effect of combat stress in the psychophysiological response of elite and non-elite soldiers. J. Med. Syst..

[8] González-González C. (2018). Actualidades en la fisiopatología del trastorno por estrés postraumático (TEPT). Salud Jalisco.

[9] (2013). American Psychiatric Association Diagnostic and Statistical Manual of Mental Disorders (DSM-5®).

[10] Hormeño-Holgado A., Nikolaidis P.T., Clemente-Suárez V.J. (2019). Psychophysiological patterns related to success in a special operation selection course. Front. Physiol.

[11] Hormeño-Holgado A.J., Clemente-Suárez V.J. (2019). Psychophysiological Monitorization in a Special Operation Selection Course. J. Med. Syst..

[12] Hormeño-Holgado A.J., Perez-Martinez M.A., Clemente-Suárez V.J. (2019). Psychophysiological response of air mobile protection teams in an air accident manoeuvre. Physiol. Behav..

[13] Bustamante-Sánchez Á., Delgado-Terán M., Clemente-Suárez V.J. (2019). Psychophysiological response of different aircrew in normobaric hypoxia training. Ergonomics.

[14] Clemente-Suárez V.J., Robles-Pérez J.J., Herrera-Mendoza K., Herrera-Tapias B., Fernández-Lucas J. (2017). Psychophysiological response and fine motor skills in high-altitude parachute jumps. High Alt. Med. Biol..

[15] Clemente-Suárez V.J., Robles-Pérez J.J., Fernández-Lucas J. (2017). Psychophysiological response in parachute jumps, the effect of experience and type of jump. Physiol. Behav..

[16] Clemente-Suárez V.J., de la Vega R., Robles-Pérez J.J., Lautenschlaeger M., Fernández-Lucas J. (2016). Experience modulates the psychophysiological response of airborne warfighters during a tactical combat parachute jump. Int. J. Psychophysiol..

[17] Clemente-Suárez V.J., Robles-Pérez J.J., Fernández-Lucas J. (2017). Psycho-physiological response in an automatic parachute jump. J. Sports Sci..

[18] Selye H. (1976). History and general outline of the stress concept. Stress in Health and Disease.

[19] Saito S. (1992). Does fatigue exist in a quantitative measurement of eye movements?. Ergonomics.

[20] Borg G.A., Noble B.J. (1974). Perceived exertion. Exerc. Sport Sci. Rev..

[21] Merchan A., Clemente-Suarez V.J. (2019). Psychophysiological modifications in an assault infantry manoeuvre using a chemical, biological, radiological and nuclear personal protective equipment. J. R. Army Med. Corps.

[22] Sánchez-Molina J., Robles-Pérez J.J., Clemente-Suárez V.J. (2018). Assessment of psychophysiological response and specific fine motor skills in combat units. J. Med. Syst..

[23] Suárez V.J.C., Pérez J.J.R. (2013). Psycho-physiological response of soldiers in urban combat. Ann. Psychol..

[24] Sánchez A.B., Herradón V.M.L., Saiz J.G., Laguna T.T., Suárez V.J.C. (2018). Psychophysiological Response of Fighter Aircraft Pilots in Normobaric Hypoxia Training. Arch. Med. Deporte..

[25] Sánchez-Molina J., Robles-Pérez J.J., Clemente-Suárez V.J. (2017). Physiological Response of a Paratrooper Unit in Urban Combat. Sports Med. Arc.

[26] Diaz-Manzano M., Fuentes J.P., Fernandez-Lucas J., Aznar-Lain S., Clemente-Suárez V.J. (2018). Higher use of techniques studied and performance in melee combat produce a higher psychophysiological stress response. Stress Health.

[27] Sánchez-Molina J., Robles-Pérez J.J., Clemente-Suárez V.J. (2019). Psychophysiological and fine motor skill differences of elite and non-elite soldiers in an urban combat simulation. Mil. Psychol..

[28] Sánchez-Molina J., Robles-Pérez J.J., Clemente-Suárez V.J. (2019). Psychophysiological and specific fine motor skill modifications in a checkpoint action. J. Med. Syst..

[29] Tornero-Aguilera J.F., Clemente-Suarez V.J. (2018). Effect of experience, equipment and fire actions in psychophysiological response and memory of soldiers in actual underground operations. Int. J. Psychophysiol..

[30] Shattock M.J., Tipton M.J. (2012). ‘Autonomic conflict’: A different way to die during cold water immersion?. J. Physiol. (Lond.).

[31] Bustamante-Sánchez Á., Clemente-Suarez V.J. (2020). Psychophysiological response in night and instrument helicopter flights. Ergonomics.

[32] Suarez V.C., Valdivielso F.N., Rave J.M.G. (2011). Changes in biochemical parameters after a 20-h ultra-endurance kayak and cycling event. Int. SportMed J..

[33] Clemente Suarez V.J., González-Ravé J.M. (2014). Four weeks of training with different aerobic workload distributions–Effect on aerobic performance. Eur. J. Sport Sci..

[34] Belinchon-deMiguel P., Clemente-Suárez V.J. (2018). Psychophysiological, body composition, biomechanical and autonomic modulation analysis procedures in an ultraendurance mountain race. J. Med. Syst..

[35] Beltrán-Velasco A.I., Bellido-Esteban A., Ruisoto-Palomera P., Clemente-Suárez V.J. (2018). Use of portable digital devices to analyze autonomic stress response in psychology objective structured clinical examination. J. Med. Syst..

[36] Vicente-Rodriguez M., Clemente-Suárez V.J. (2020). Psychophysiological anxiety response of a rescue helicopter crew in a crane rescue manoeuvre. BMJ Mil. Health.

[37] Hormeño-Holgado A.J., Clemente-Suárez V.J. (2019). Effect of different combat jet manoeuvres in the psychophysiological response of professional pilots. Physiol. Behav..

